# An R2R3 MYB transcription factor determines red petal colour in an *Actinidia* (kiwifruit) hybrid population

**DOI:** 10.1186/1471-2164-14-28

**Published:** 2013-01-16

**Authors:** Lena G Fraser, Alan G Seal, Mirco Montefiori, Tony K McGhie, Gianna K Tsang, Paul M Datson, Elena Hilario, Hinga E Marsh, Juanita K Dunn, Roger P Hellens, Kevin M Davies, Mark A McNeilage, H Nihal De Silva, Andrew C Allan

**Affiliations:** 1 The New Zealand Institute for Plant & Food Research Limited, 120 Mt. Albert Road, Auckland, 1142, New Zealand; 2 The New Zealand Institute for Plant & Food Research Limited, 412 No. 1 Road, RD 2, Te Puke, 3182, New Zealand; 3 The New Zealand Institute for Plant & Food Research Limited, Fitzherbert Science Centre, Batchelar Road, Palmerston North, 4474, New Zealand

**Keywords:** *Actinidia*, Kiwifruit, Anthocyanin, MYB, Transcription factor, Colour

## Abstract

**Background:**

Red colour in kiwifruit results from the presence of anthocyanin pigments. Their expression, however, is complex, and varies among genotypes, species, tissues and environments. An understanding of the biosynthesis, physiology and genetics of the anthocyanins involved, and the control of their expression in different tissues, is required. A complex, the MBW complex, consisting of R2R3-MYB and bHLH transcription factors together with a WD-repeat protein, activates anthocyanin 3-*O*-galactosyltransferase (*F3GT1*) to produce anthocyanins. We examined the expression and genetic control of anthocyanins in flowers of *Actinidia* hybrid families segregating for red and white petal colour.

**Results:**

Four inter-related backcross families between *Actinidia chinensis* Planch. var. *chinensis* and *Actinidia eriantha* Benth. were identified that segregated 1:1 for red or white petal colour. Flower pigments consisted of five known anthocyanins (two delphinidin-based and three cyanidin-based) and three unknowns. Intensity and hue differed in red petals from pale pink to deep magenta, and while intensity of colour increased with total concentration of anthocyanin, no association was found between any particular anthocyanin data and hue. Real time qPCR demonstrated that an R2R3 MYB, *MYB110a,* was expressed at significant levels in red-petalled progeny, but not in individuals with white petals.

A microsatellite marker was developed that identified alleles that segregated with red petal colour, but not with ovary, stamen filament, or fruit flesh colour in these families. The marker mapped to chromosome 10 in *Actinidia*.

The white petal phenotype was complemented by syringing *Agrobacterium tumefaciens* carrying *Actinidia 35S*::*MYB110a* into the petal tissue. Red pigments developed in white petals both with, and without, co-transformation with *Actinidia* bHLH partners. *MYB110a* was shown to directly activate *Actinidia F3GT1* in transient assays.

**Conclusions:**

The transcription factor, *MYB110a*, regulates anthocyanin production in petals in this hybrid population, but not in other flower tissues or mature fruit. The identification of delphinidin-based anthocyanins in these flowers provides candidates for colour enhancement in novel fruits.

## Background

Flavonoids are a large, diverse group of plant phenolic compounds involved in a variety of biological responses to the biotic and abiotic environment, such as disease resistance, seed dormancy, pigmentation of flowers and fruits, protection against UV-B damage, and reduction of insect and mammalian herbivory [1,2]. The anthocyanin pigments that are produced by a branch of the flavonoid pathway are important as insect and animal attractants, for pollination and seed dispersal. However, they are also of increasing importance as quality traits in commercial fruit crops, not only for the colours they produce but also for their potential human health benefits. A wide range of cell culture experiments, animal trials and epidemiological studies have linked intake of anthocyanins with reduced risk for a range of health problems, including heart disease, cancer, diabetes and degenerative conditions such as Alzheimer's disease [3-6].

As flavonoids contribute to many different functions at various times in a plant, a complex system of regulation is required to direct temporal and spatial production in response to developmental and environmental stimuli. The primary point of regulation of flavonoid production occurs at the transcriptional level, through activation or repression of the biosynthetic genes by a conserved group of transcription factors (TFs). In all plants studied to date, activation of the anthocyanin pathway is through a 'MBW complex' consisting of R2R3-MYB and bHLH TFs and a WD-repeat (WDR) protein that may facilitate protein-protein interactions [7-9]. In addition to the MYBs that are part of the activation complex, distinct R2R3- and R3-MYBs with a repressive action have been identified that may allow for feedback modulation of the amount of anthocyanin produced [10-14].

Based on previous studies, it seems likely that variation in activity of the R2R3-MYB genes is key to determining the spatial and temporal patterning of anthocyanin production in most plant species [15,16]. The R2R3-MYB and bHLH families of transcription factors are two of the largest in plants, with 139 and 162 members respectively, in *Arabidopsis*[17]. The R2R3-MYBs that activate anthocyanin biosynthesis have diverged during evolution to control anthocyanin production only in response to specific stimuli or with a high degree of spatial specificity. R2R3-MYB genes have been identified that are associated with activating anthocyanin biosynthesis in flowers, fruits, or vegetative tissues [11,15,16,18-20]. Genetic changes affecting individual members of the R2R3-MYB or bHLH gene families can thus result in tissue-specific changes in anthocyanin production. The identification of the R2R3-MYB and bHLH genes associated with anthocyanin production in a particular species is therefore an important step towards elucidating the genetic control of pigmentation in that species.

Petals of the kiwifruit species *Actinidia chinensis* Planch. var. *chinensis* are white or cream, shading to apricot during senescence, while those of *A. eriantha* Benth. are typically pink or red, although a white-flowered form has been found in China [21]. F_1_ hybrids from crosses between tetraploid *A. chinensis* var. *chinensis* and *A. eriantha* are known to produce red flowers [22,23]. Zhang et al. (2010) studied the segregation of flower colour among 22 seedlings from a cross between hexaploid *A. chinensis* var. *deliciosa* and a diploid male F_1_ hybrid between diploid *A. eriantha* and tetraploid *A. chinensis* var. *chinensis*[24]. The seedlings could be broadly classified into those with red and those with white-coloured petals, suggesting the action of a small number of genes. However, because of the complexity of the pedigree, the small number of seedlings and the presence of intermediate types with red and white petals, no firm conclusions could be drawn about the inheritance of petal colour. Fan et al. (2004) studied the flower colour of 26 seedlings obtained from a cross between hexaploid *A. chinensis* var. *deliciosa* and diploid *A. eriantha*[25]. Female seedlings all had white flowers while the male seedlings had red, white or peach and white flowers. The authors reached no conclusion regarding inheritance of petal colour.

In this paper, we report the results from a study of petal colour in two families of *(A. eriantha* x *A. chinensis)* x *A. chinensis var. chinensis* (EACK x CK) hybrids and two families of A. *chinensis* var. *chinensis* x *A. eriantha*) x *A. chinensis* var. *chinensis* (CKEA x CK) hybrids (Additional files 1, 2). The scale of the population and its genetic structure, and the ability to access a large Expressed Sequence Tag (EST) database for *Actinidia* species in which to seek candidate regulatory genes, enabled us to examine the genetic control of petal colour in these hybrids [26].

## Results

### The phenotyping of petal colour of the four backcross families

The ratio of vines with red or white petals in each of the four families was not significantly different from 1:1, and there were no significant differences in petal colour ratio among families (*P* = 0.382 – 0.749) (Table 1), or between sexes within families (*P* = 0.109 – 1.000). This result would suggest that, in these hybrid families, petal colour inheritance is a monogenic dominant trait.

**Table 1:** **Phenotypes of four *Actinidia *hybrid backcross families**

**Family**	**Red male**	**White male**	**Red female**	**White female**	**Total Red**	**Total White**	***P *value H_O: ratio = 1:1**
1	26	24	16	22	42	46	0.749
2	12	14	12	16	24	30	0.497
3	10	14	10	13	20	27	0.382
4	29	25	19	17	48	42	0.598
Total	77	77	57	68	134	145	0.550

Segregation for petal colour and gender in two families of backcross seedlings of (*A. eriantha* x *A. chinensis* var. *chinensis*) x *A. chinensis* var. *chinensis* (families 1 and 2), and two families of backcross seedlings of (*A. chinensis* var. *chinensis* x *A. eriantha*) x *A. chinensis* var. *chinensis* (families 3 and 4). The ratio of red to white petal colour of approximately 1:1 suggests that in petals of these hybrid families colour inheritance is a monogenic dominant trait.

**Figure 1:**
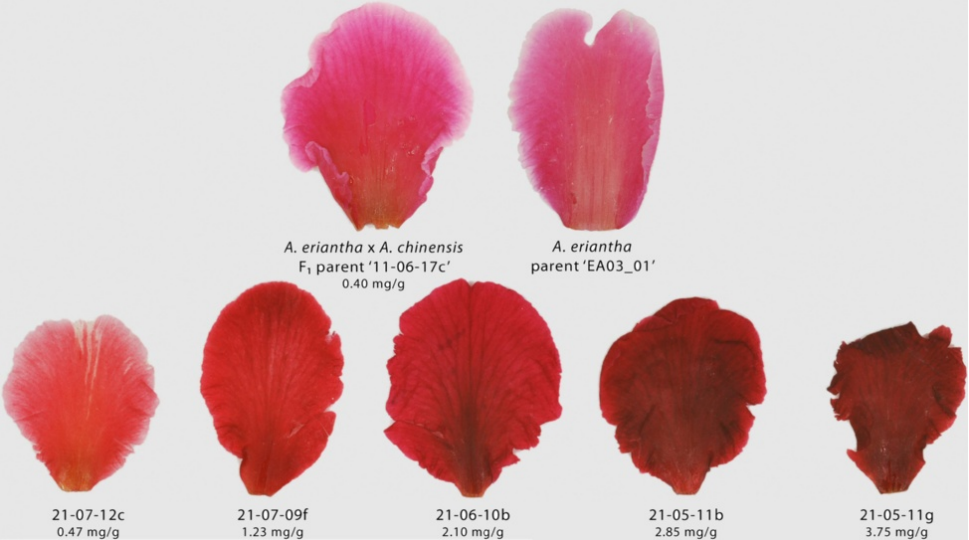
**Range of colour intensities in the petals of progeny compared with parents.** The progeny of all four *Actinidia* families making up the experimental population showed a range of intensities in anthocyanin expression in their petals. While some red petal genotypes had flower petals that were paler than their F_1_ hybrid parent, or *A. eriantha*, many had much darker red petals. There was a constancy of petal colour within vines. The concentrations of colour compounds were recorded as mg.g^-1^ fresh weight of petal tissue. Each progeny genotype was identified by its field position with orchard block number, row number and bay position giving a unique identifier. Parental genotypes may be identified by their field position as described, or their accession number in the germplasm resource.

Among progeny vines with red petals, some variation was observed in the intensity of red colouration between vines and many had much darker red petals than those of their F_1_ hybrid parent or *A. eriantha* grandparent (Figure 1). Intensity of colour usually declined from the base of the petal to the fringe. However, there was very little variation between flowers within each vine.

Of 101 vines scored for the colour of the ovary pericarp tissue, only 11 had red colouration, of which four showed intense red colour. These 11 vines all belonged to the same family (family 1) and thus had the same parentage. Ovary colour was independent of petal colour. Stamen filament colour was also independent of petal and ovary colour. Red filaments were found with both red or white petals, and red or green ovaries, in all combinations in female progeny, and with red or white petals in males (Additional file 3).

Of 88 female vines screened for fruit flesh colour, only four had fruit with some red colouration and the red colour was faint and confined to the area around the core towards the pedicel end of the fruit. There was no obvious association between red colour in the ovary, petals, filaments or fruit flesh.

### Quantification and identification of anthocyanins and flavonols extracted from red petals of individual genotypes

**Figure 2:**
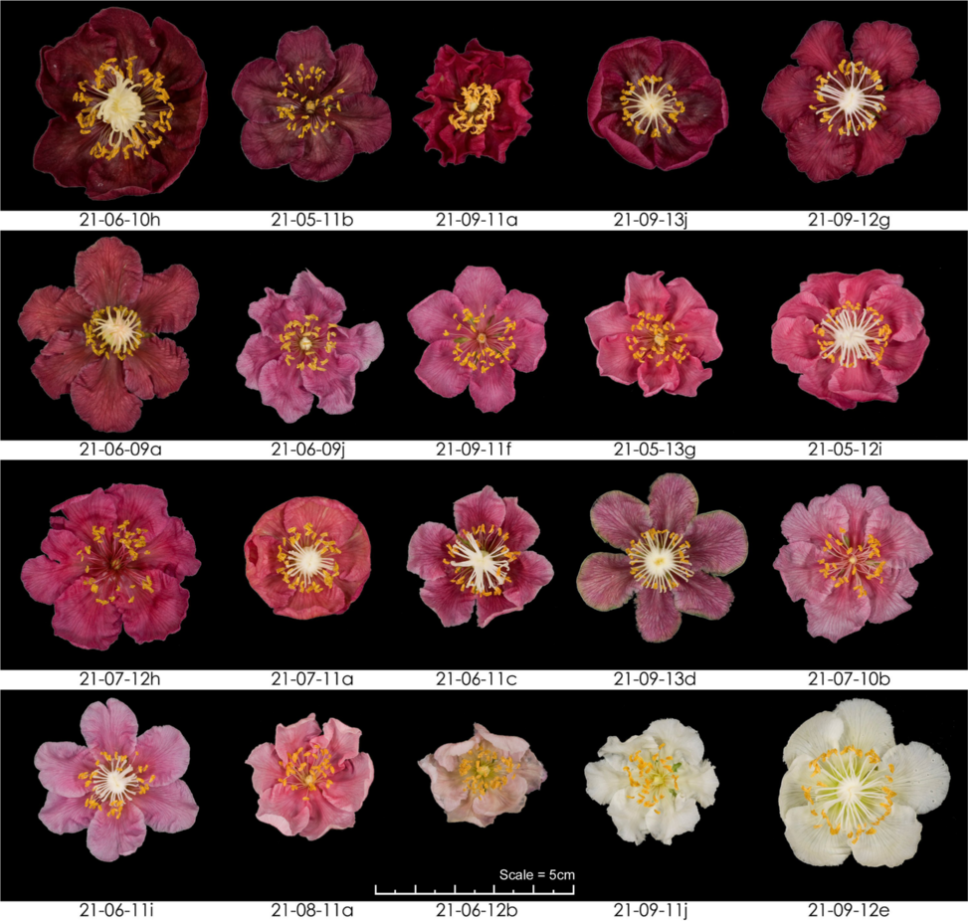
**Colour intensity and hue variation of petals among progeny genotypes.***Actinidia* progeny segregating for red and white petal colour within all four families showed significant variation among genotypes in both colour intensity and hue of the flower petals. The red flowers ranged from a pale pink to a deep magenta. The unique genotype identity of field position is shown below a typical flower of each.

**Figure 3:**
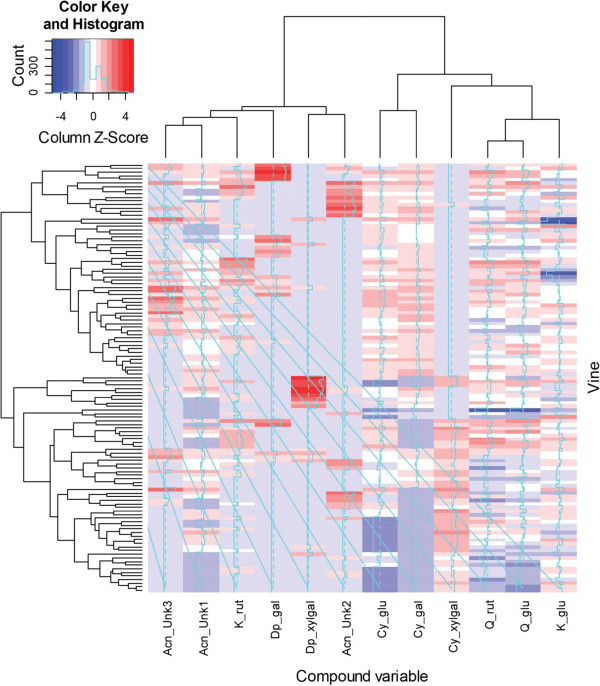
**Analysis of anthocyanin and flavonol data.** Heatmap of concentrations of 12 pigment compounds measured on 138 vines of the hybrid F_2_ backcross families between (*Actinidia eriantha* x *A. chinensis* var. *chinensis*) and *A. chinensis var. chinensis.* The data displayed on a colour scale are the normalised (Z scores) log^2^ values of the raw data. The dendrograms corresponding to rows (i.e., vines) and columns (i.e., compound variables) have been constructed by hierarchical clustering. The compounds measured were the anthocyanins delphinidin 3-*O*-(xylosyl)galactoside, delphinidin 3-*O*-galactoside, cyanidin 3-*O*-(xylosyl)galactoside, cyanidin 3-*O*-galactoside, cyanidin 3-*O*-glucoside and three unidentified anthocyanins, and the flavonols quercetin-rutinoside, quercetin-glucoside, kaemferol-glucoside and kaemferol-rutinoside.

In all four families there was variation in hue among the red-petalled genotypes (Figure 2). As dihydroxyflavonols are substrates for production of flavonols, pro-anthocyanidins and anthocyanins, and because flavonols are known to act as co-pigments affecting hue, the amounts of these compounds were assayed at the same time as the anthocyanins (Figure 3, Additional file 4).

**Figure 4:**
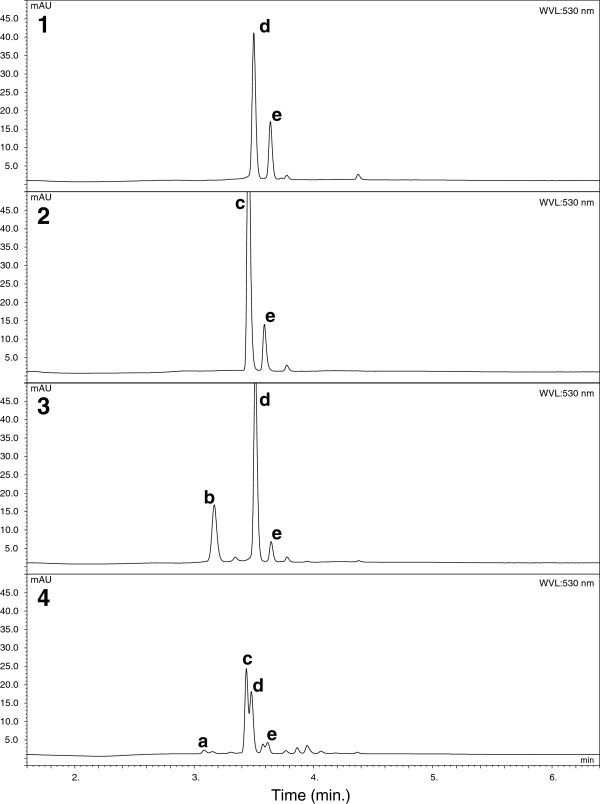
**Anthocyanin profiles of red petals of genotypes segregating for this phenotype.** Analysis of anthocyanins in red *Actinidia* petals by Ultra High Performance Liquid Chromatography (UHPLC)(WVL 530 nm) identified four patterns that commonly occurred. Traces 1 to 4 show the anthocyanins present, and their concentrations, in the sample as: a - delphinidin 3-*O*-(xylosyl)galactoside; b - delphinidin 3-*O*-galactoside; c – cyanidin 3-*O*-(xylosyl)galactoside; d - cyanidin 3-*O*-galactoside; e – cyanidin 3-*O*-glucoside. Trace 1 is dominated by cyanidin 3-*O*-galactoside/cyanidin 3-*O*-glucoside. Cyanidin 3-*O*-(xylosyl)galactoside is the most plentiful anthocyanin present in trace 2, while trace 3 shows a significant concentration of delphinidin 3-*O*-galactoside together with cyanidin 3-*O*-galactoside/cyanidin 3-*O*-glucoside. Trace 4 shows a pattern of multiple anthocyanins with delphinidin 3-*O*-(xylosyl)galactoside, delphinidin 3-*O*-galactoside, cyanidin 3-*O*-(xylosyl)galactoside, cyanidin 3-*O*-galactoside and cyanidin 3-*O*-glucoside all present.

Eight anthocyanins were detected in red petal tissue. Two of these were classified as delphinidin-based and three as cyanidin-based, while the identities of three anthocyanins that were present in small quantities were not determined (Anthocyanin Unknown, Acn-Unk1 to Acn-Unk3). The concentration of all compounds was recorded as mg.g^-1^ fresh weight of petal tissue. The delphinidin-based anthocyanins, delphinidin 3-*O*-(xylosyl)galactoside (dp-xylgal) and delphinidin 3-*O*-galactoside (dp-gal), were present in only 40 of the 134 genotypes sampled, and family 2 progeny did not contain delphinidin compounds. Families 1, 3 and 4 had 11 genotypes with dp-xylgal only and 24 genotypes with dp-gal only, while family 1 also had five genotypes with both delphinidins (Additional file 4). The three cyanidin-based anthocyanins identified were cyanidin 3-*O*-(xylosyl)galactoside (cy-xylgal), cyanidin 3-*O*-galactoside (cy-gal) and cyanidin 3-*O*-glucoside (cy-glu). When summed, anthocyanins ranged in concentration from 0.11 mg.g^-1^ to 3.73 mg.g^-1^, with the higher concentrations reflected in the depth of petal colour. Cy-gal and cy-glu were the two most commonly occurring identified anthocyanins, and Acn-Unk1 also occurred in most genotypes but in very low concentrations from 0.01 to 0.08 mg.g^-1^. Cy-gal and cy-glu generally occurred in the same genotypes, but the concentrations of cy-glu were significantly lower than those of cy-gal (Additional file 4). Genotypes were recorded as having from one anthocyanin present, either cy-xylgal or cy-gal, to having up to all eight anthocyanins present. Among the chromatograms recorded, four patterns of anthocyanin concentration were found (Figure 4). In Figure 4 – traces 1, 2 and 3 respectively, the anthocyanins identified in greatest concentration were 1: cy-gal and cy-glu; 2: cy-xylgal and cy-glu; and 3: cy-gal and dp-gal. In the fourth pattern multiple anthocyanins were present, with cy-xylgal and cy-gal dominating the anthocyanin profiles.

Four flavonols were assayed as potential co-pigments. Quercetin-rutinoside (q-rut), quercetin-glucoside (q-glu) and kaemferol-glucoside (k-glu) were present in all genotypes. Concentrations ranged from 0.07 mg.g^-1^ to 2.02 (q-rut), 3.39 (q-glu) and 6.13 (k-glu) mg.g^-1^ of petal tissue. Kaemferol-rutinoside (k-rut) was present in 60 of the 134 genotypes, and at much lower concentrations between 0.02 and 0.33 mg.g^-1^. There was no apparent association between the concentration of flavonol present and the perceived hue of the petals.

### Identification of candidate anthocyanin regulatory factors

Previous research has shown the key role of the MYB/bHLH/WDR complex in regulation of the flavonoid pathway, and, in particular a subgroup of R2R3-MYB characterized by the presence of the bHLH interacting signature ([DE]Lx_2_[RK]x_3_Lx_6_Lx_3_R) in the R2R3 domain, and a C-terminus KPRPR[S/T]F motif typical of anthocyanin regulators [27,28]. From an extensive EST database from different species and tissues of *Actinidia*[26], two R2R3-MYBs were identified, *MYB10* and *MYB110a*, with both these motifs [29]. Further sequencing of kiwifruit genomic sequences led to the identification of a third R2R3-MYB (termed *MYB110b*), almost identical in the coding sequence region to *MYB110a*, but which proved, through expression studies, to be a different gene and not an allelic variation of *MYB110a*, and assigned to a different locus by genetic mapping.

**Figure 5:**
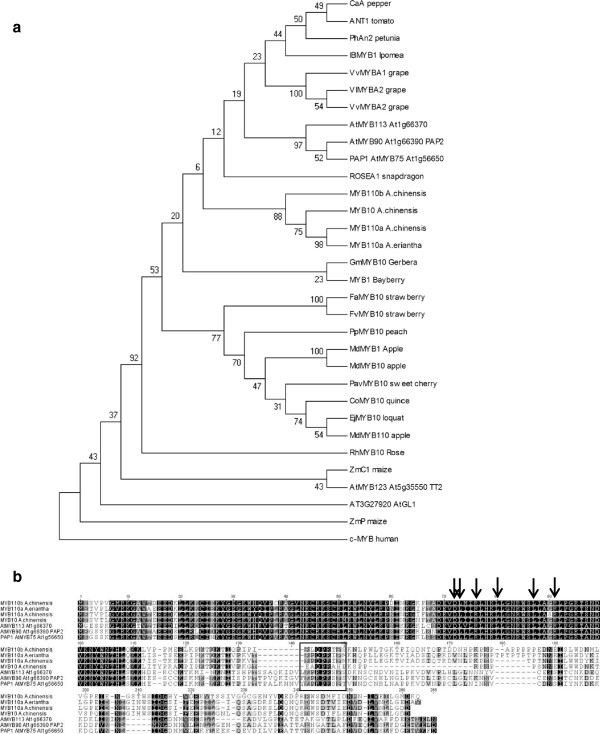
**Phylogeny of *A. chinensis *and *A. eriantha *MYB genes evaluated from protein sequence. (a)**. Phylogeny of MYB proteins implicated in anthocyanin regulation in various genera, and the positions assumed by the *MYB10* and *MYB110* of *Actinidia chinensis* var. *chinensis* and *A. eriantha*. Phylogenetic and molecular evolutionary analysis was conducted using MEGA version 4.0.2 [38] (using minimum evolution phylogeny test and 1000 bootstrap replicates). GenBank numbers; RhMYB10 (ABX79949), ZmP (AF292540), ZmC1 (Y15219), IbMYB1 (AB576767), GmMYB10 (ACM62751), ROSEA1 (DQ275529), MYB1 bayberry (GQ340767), VvMYBA2 (ABL14065), VlMYBA2 (BAC07540), VvMYBA1 (AB242302), ANT1 (AY348870), PhAn2 (EF423868), CaA (AJ608992), FvMYB10 (ABX79948), FaMYB10 (ABX79947), PpMYB10 (ABX79945), PavMYB10 (ABX71493), MdMYB110 (EB710109), EjMYB10 (ABX71484), CoMYB10 (ABX71483), MdMYB10 (ACQ45201), MdMYB1 (DQ886414) and c-MYB (AAB49039). **(b)**. Protein sequence data from which phylogenetic relationships of MYB genes were evaluated. Sequences were aligned using Clustal W (opening = 15, extension-0.3) in Vector NTI9.0. Arrows indicate the amino acid signature motif ([DE]Lx_2_[RK]x_3_Lx_6_Lx_3_R) in the R2R3 domain, which allows interaction with a bHLH partner, and the box shows the C-terminus motif typical of anthocyanin-related regulators.

In a phylogenetic tree based on the alignment of the deduced amino acid sequences, the three MYB genes: *MYB10*, *MYB110a*, *MYB110b*, were grouped with the other R2R3-MYB genes shown to be involved in the anthocyanin regulatory process in several plant species (Figures 5a, 5b).

### Gel and capillary array electrophoresis of two *MYB110* candidate genes in families segregating for petal colour

The sequence of each of the *MYB110* genes in this study was examined and PCR primer pairs were designed to create microsatellite markers that would identify each gene. *MYB110a* was represented by marker Ke923 and *MYB110b* by marker Ke701. The PCR products amplified by the markers of both the *MYB110* candidate genes were examined by gel electrophoresis with a sample set of both red and white-petalled progeny from the interspecific backcross. Both markers were seen to be polymorphic, with particular bands segregating with red petal colour.

**Figure 6:**
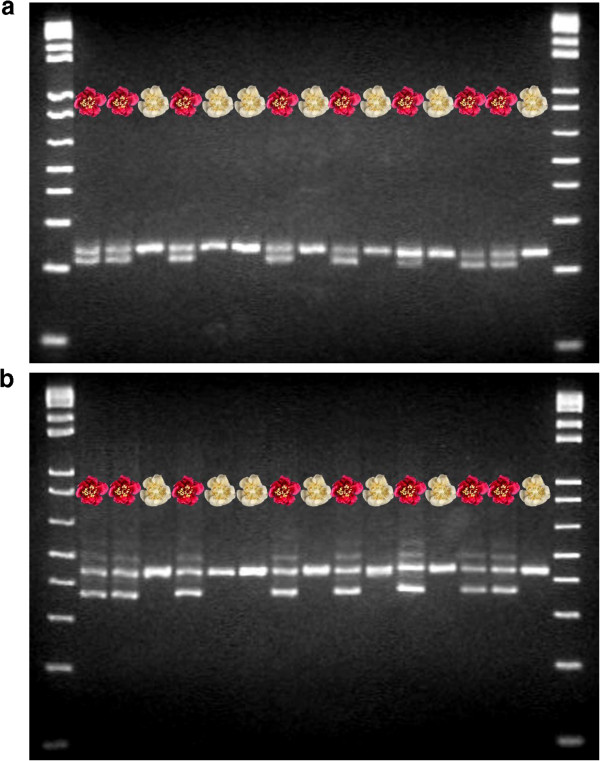
**Gel electrophoresis banding patterns identify red-petal progeny.** Progeny of four interspecific hybrid families between (*Actinidia chinensis* var. *chinensis* x *Actinidia eriantha*) x *Actinidia chinensis* var. *chinensis* had either red or white-petalled flowers. Gel electrophoresis of the PCR products amplified with primers to the *MYB110* genes showed that the genotypes could be distinguished on the basis of their banding patterns. With *MYB110a*, (primer pair Ke923) genotypes with red petal colour all had two clear bands, while genotypes with white petal colour had a single band (Figure 6**a**). Primer pair Ke701, specific to *MYB110b*, also segregated with red petal colour (Figure 6**b**). In this instance the red petal phenotype had three bands while the white petal genotype had one band. Both gels were run through a 2.75% agarose gel with a 1Kb + size ladder.

A single band for *MYB110a* was amplified in white petal types using Ke923 primers, while a second band clearly amplified in all red petal samples (Figure 6a). Capillary array electrophoresis with these primers revealed products of two sizes amplified in the available parents of the crosses made to produce the F_2_ backcross families (Table 2). The red-petalled *A. eriantha* or *A. eriantha* x *A. chinensis* var. *chinensis* parents were found to carry alleles corresponding to PCR products of sizes 209 bases (allele 209), and 228 bases (allele 228), and the presence of allele 209 conferred the red phenotype for petal colour. The male parent, *A. chinensis* CK15_02, of the F_2_ population was homozygous and carried allele 228. This simple allelic pattern in the parents gave rise to red-petalled progeny with alleles 209 and 228, and white-petalled progeny that were homozygous for allele 228.

**Table 2:** **Allelic composition of the parents and progeny of the F**_**2 **_**hybrid backcross**

**Progeny sample**	**G**	**Fam**	**Petal colour**	**Ke923 (*MYB110a*) alleles**		**Ke701 (*MYB110b*) alleles**									
				**1**	**2**	**1**	**2**	**3**	**4**	**5**	**6**	**7**	**8**	**9**	**10**
21-05-08g	m	1	red	209	228		361						426		
21-05-08j	m	1	red	209	228		361						426		
21-07-09c	f	1	red	209	228		361						426		
21-06-10h	f	1	red	209	228		361							427	
21-07-09f	m	1	red	209	228		361							427	
21-07-09g	f	1	red	209	228		361							427	
21-07-09b	f	1	white		228						410		426		
21-07-12e	m	1	white		228						410		426		
21-08-08h	f	1	white		228						410		426		
21-07-09a	m	1	white		228						410			427	
21-07-12b	f	1	white		228						410			427	
21-08-08b	f	1	white		228						410			427	
21-05-09f	m	2	red	209	228	359							426		
21-07-13d	m	2	red	209	228	359							426		
21-06-11i	f	2	red	209	228	359								427	
21-07-13e	f	2	red	209	228	359								427	
21-08-11a	m	2	red	209	228	359								427	
21-05-09b	m	2	white		228								426		432
21-05-09e	f	2	white		228								426		432
21-05-09h	m	2	white		228								426		432
21-05-09g	f	2	white		228									427	432
21-07-13g	f	2	white		228									427	432
21-07-14g	m	2	white		228									427	432
21-05-12i	f	3	red	209	228	359							426		
21-07-11c	m	3	red	209	228	359							426		
21-09-10c	f	3	red	209	228	359							426		
21-05-12a	f	3	red	209	228	359								427	
21-05-12g	f	3	red	209	228	359								427	
21-07-11b	m	3	red	209	228	359								427	
21-06-13a	m	3	white		228								426		
21-08-09h	f	3	white		228									427	
21-06-08a	f	4	red	209	228	359							426		
21-06-12b	m	4	red	209	228	359							426		
21-07-08h	f	4	red	209	228	359							426		
21-07-10b	m	4	red	209	228	359							426		
21-09-11f	m	4	red	209	228	359							426		
21-06-08h	f	4	red	209	228	359								427	
21-07-10h	f	4	red	209	228	359								427	
21-09-09a	f	4	red	209	228	359								427	
21-09-09d	m	4	red	209	228	359								427	
21-07-08a	m	4	white		228								426		
21-07-08b	f	4	white		228								426		
21-07-10e	f	4	white		228								426		
21-07-10f	m	4	white		228								426		
Parents															
CK01_03	f		white	nd							410	413			
CK01F2	f		white		228			406	408						
CK15_01	m		white		228								426		
CK15_02	m		white		228								426	427	
CK21_01	f		white		228					409					432
EA03_01	f		red	209		359	361								
EA04_03	f		red	209	228	359									
EA07_R22	m		red	209	228	nd									
Hort16A	f		white		228			406					426		
11-06-17c	f	1	red	209	228		361				410				
11-06-16e	f	2	red	209	228	359									432
11-06-15d	f	3	red	209	228	359							426		
11-06-15c	f	4	red	209	228	359							426		

Analysis by capillary electrophoresis of PCR products with the *MYB110* gene markers showed that both *MYB110a* and *MYB110b* carried alleles that segregated with red petal colour. In all the parents available for analysis, and progeny, only two alleles were amplified with *MYB110a* marker primers. Red-petalled *Actinidia* genotypes carried alleles represented by PCR products sized 209 (allele 209) and 228 (allele 228), while white-petalled genotypes were homozygous for allele 228. Analysis with *MYB110b* showed a total of ten alleles amplified in parents. Of these ten alleles, only six were found in the F_2_ backcross progeny. Two of the six alleles, alleles 359 and 361, segregated with red petal colour. The presence of either allele gave the red phenotype.

Gel electrophoresis with the primer pair representing *MYB110b* demonstrated that white petal genotypes gave a single band while two bands, with a third minor band, were present in all red petal samples (Figure 6b). With capillary array electrophoresis, a total of ten alleles produced amplification products in the available parents of the families (Table 2). Of the ten alleles shown in parental genotypes, only six were recorded in the progeny of the four families. Of these six alleles, two were found to segregate with red colour, alleles 359 and 361 (Table 2). Family 1 carried allele 361 while families 2, 3 and 4 all carried allele 359.

### Mapping of *MYB110a* (Ke923), *MYB110b* (Ke701) and *MYB10* (Ke922)

Three sets of primers were used to amplify markers within *MYB10*, *MYB110a* and *MYB110b* gene regions. Marker Ke922, designed for the *MYB10* gene, amplified a female informative marker [a(281)b(275) x a(281)a(281)] in a diploid *A. chinensis* var. *chinensis* mapping population (see Fraser et al. 2009 for mapping details [30]). Inclusion of this marker into a dataset of microsatellite markers from this population, and using JoinMap 3.0 to group and order markers, linked the gene to linkage group 20. Marker Ke701, designed for the *MYB110b* gene, amplified a fully informative marker (a(413)b(415) x c(426)d(427)) in the diploid *A. chinensis* var. *chinensis* mapping population and genetic mapping linked this gene to linkage group 10. Marker Ke923, designed for the *MYB110a* gene, amplified a non-polymorphic marker (aaxaa) in the diploid *A. chinensis* mapping family, so the position of this gene within the genome was unable to be determined by genetic mapping in *A. chinensis*. However, in the (*A. chinensis* var. *chinensis* x *A. eriantha*) x *A. chinensis* var. *chinensis* hybrids, marker Ke701 was fully informative and Ke923 was female informative and the two markers were tightly linked together and also tightly linked with the red petal phenotype (Table 2).

### *MYB110a* expression correlates with anthocyanin synthesis in flower petals

Cyanidin 3-*O*-galactoside is an abundant anthocyanin in red petals and is required for the formation of cyanidin 3-*O*-xylogalactose (Figure 3). *F3GT1* is an anthocyanin 3-*O*-galactosyltransferase previously described in fruit of *A. chinensis* that glycosylates cyanidin 3-*O*-galactoside *in vitro*[31]. By quantitative PCR (qPCR) we analysed the level of transcript of *F3GT1* in four different genotypes, of which two (21-05-08a and 21-05-11f) had white petals, and two (21-08-12d and 21-05-10h) had bright red petals, at two different stages of flower development: calyx split and full bloom.

**Figure 7:**
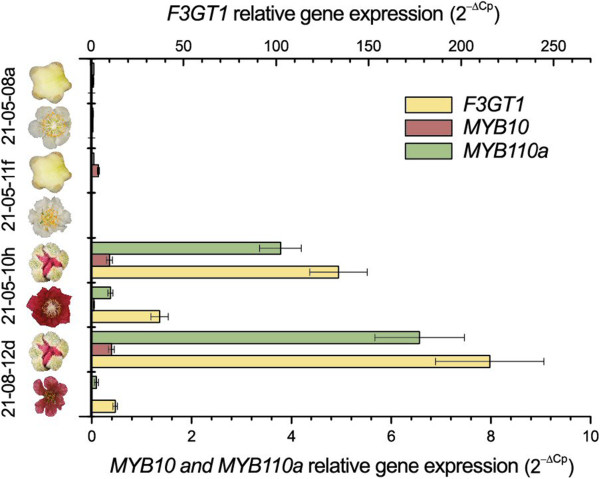
**qPCR – Relative expression of *F3GT1*, *MYB10 *and *MYB110a *in petals.** Gene expression analysis of *MYB10* (primer set Ke922), *MYB110a* (primer set Ke923) and *F3GT1* (Flavonoid 3-O-galactosyltransferase) in petals of white and red flowers of an interspecific *Actinidia* hybrid (*A. chinensis* var. *chinensis* x *A. eriantha*) x *A. chinensis* var. *chinensis* at two stages of flower development: calyx split and open flower. Error bars are SE for four technical replicates of each genotype.

Transcript levels of *F3GT1* were high in 21-08-12d and 21-05-10h during flower development, but barely detectable in 21-05-08a and 21-05-11f (Figure 7). This result correlates with the degree of pigmentation of the petals. In the genotypes with red petals, the level of transcript of *F3GT1* was higher at the calyx split stage of development and decreased as the flower developed to full bloom. In the white flowered genotypes, the level of transcript was barely detectable at either stage tested. Transcript levels of both *MYB110a* and *MYB10* were higher at the early stage of calyx split in the two red petal genotypes, and both were seen to decline at the later stage of full bloom (Figure 7). *MYB110a* showed a high level of transcript in the red petals, while *MYB10* was expressed, but at a much lower level. cDNA sequence analysis of the expressed transcript confirmed the identification as *MYB110a*. Primers specific for *MYB110b* did not amplify any transcript, indicating that *MYB110b* is not expressed in petals.

In the white petals the level of *MYB110a* transcript was barely detectable at any stage. *MYB110a* expression appeared to be strongly correlated with *F3GT1* expression, being present in the same tissue and showing the same pattern of expression. Both were highly expressed at calyx split, with expression declining at full bloom. This strong link between the transcript level of *MYB110a* and *F3GT1* suggests the involvement of *MYB110a* in the regulation of the transcription of *F3GT1*, and of the anthocyanin biosynthesis pathway in the petals of *A. eriantha,* and of the studied population.

### *MYB110a* regulates *F3GT1* expression and anthocyanin biosynthesis

**Figure 8:**
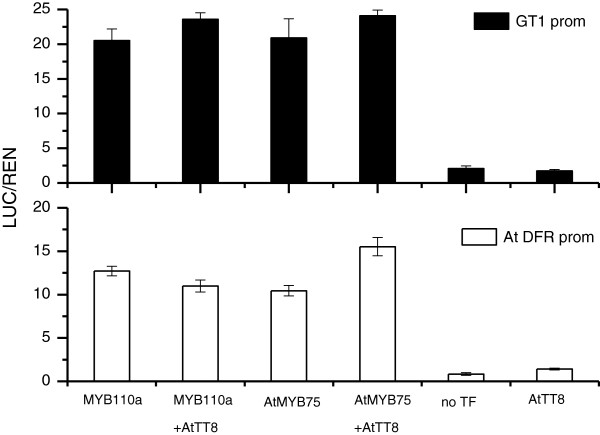
**Trans-activation of the *F3GT1 *and *AtDFR *promoters by *MYB110a *and *AtMYB75 *in a dual luciferase transient assay.** Leaves of *Nicotiana benthamiana* were infiltrated with *F3GT1* promoter-*LUC* or *AtDFR* promoter-*LUC* fusions on their own or co-infiltrated with *35S*::*MYB110a*, *35S*::*AtMYB75* or with *35S*::*AtTT8*. Luminescence of *LUC* and *REN* was measured 3 d later and expressed as a ratio of *LUC* to *REN*. Data are presented as means (± SE) of four biological replicates of each transgene combination.

To assess the ability of *MYB110a* to regulate gene expression of the anthocyanin pathway in *Actinidia* flower petals, it was determined if *MYB110a* was able to trans-activate the *Actinidia F3GT1* promoter in a transient luciferase assay performed in *Nicotiana benthamiana* leaves. The luciferase assay showed that the *F3GT1* promoter was strongly activated by *MYB110a* (Figure 8). Co-transformation of *MYB110a* with the *Arabidopsis* bHLH *TT8* slightly increased luciferase activity. A similar result was obtained when the *Arabidopsis* R2R3MYB *AtMYB75 (PAP1)* was used, while no activation occurred when the bHLH *AtTT8* alone was tested (Figure 8). When an Arabidopsis promoter *AtDFR*[32], fused to luciferase was used, *MYB110a* showed strong activation with and without *AtTT8*, while *AtMYB75* was significantly stimulated by the presence of *AtTT8*. This suggests that *MYB110a* was either able to recruit endogenous tobacco bHLH and WDR and form the MBW complex to induce promoter activation, or other co-factors are involved.

**Figure 9:**
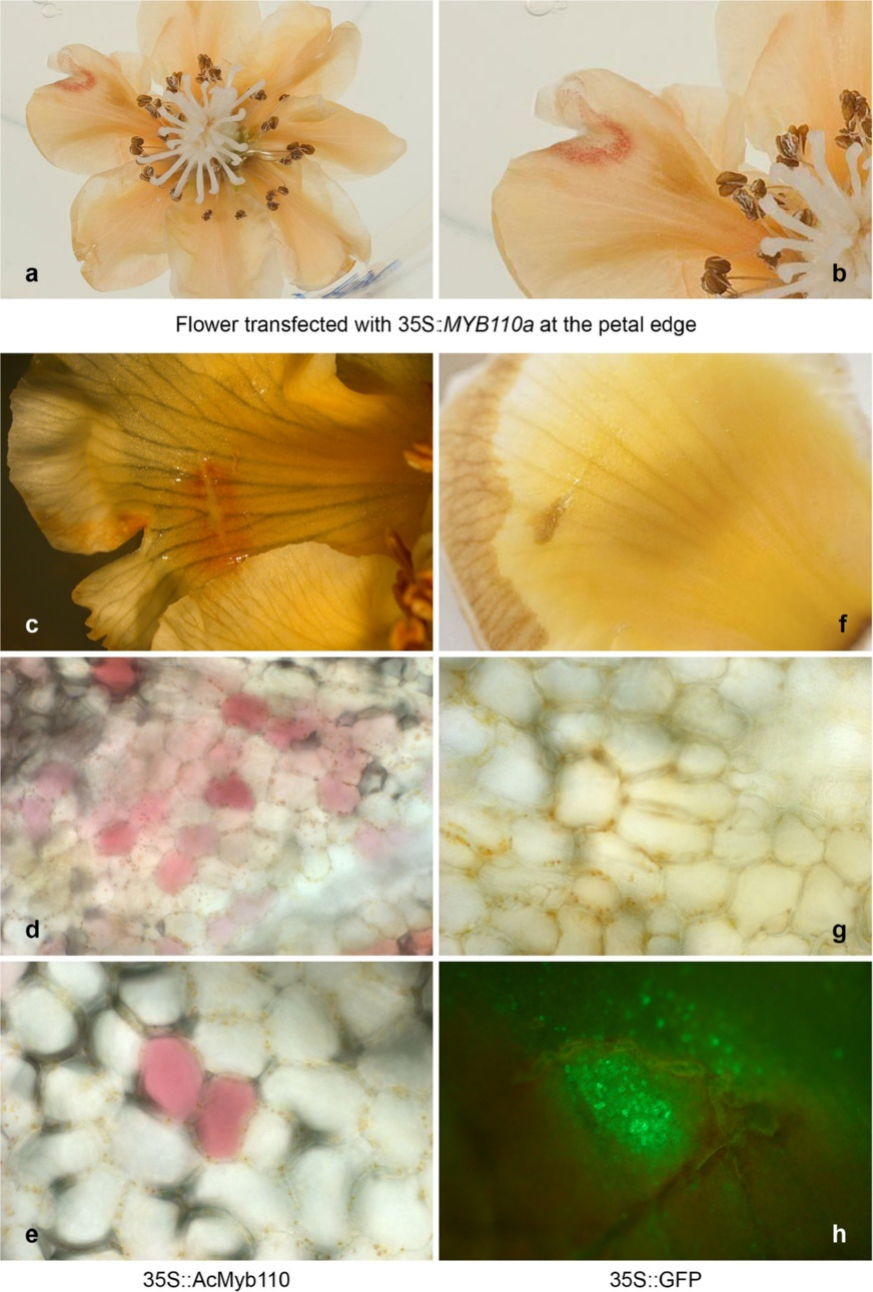
**Complementation of the white petal phenotype in a transient assay by over-expression of *MYB110a*.** White flowers of a progeny genotype (21-05-09e) of the F_2_ backcross (*Actinidia chinensis* var. *chinensis* x *Actinidia eriantha*) x *Actinidia chinensis* var. *chinensis* hybrid population were syringed at a cut surface and kept on agar for four days. A flower was transfected with *35S*::*MYB110a* at the petal edge. In the surrounding area of the infiltration, over-expression of *MYB110a* complemented the white phenotype and restored the ability to synthesise anthocyanin in petals. The white flowers followed their natural senescence pathway and coloured to light apricot during the four-day experiment. Microscopy showed that within the area of infiltration single cells within the petal transfected with *35S*::*MYB110a* were highly pigmented (magnification x10 upper, and x40 lower panel), and the pigment was contained within the cells (Figure 9**c**, 9**d**, 9**e**). When the petal was transfected with *35S*::*GFP* as control, the cells did not show any anthocyanin accumulation, light (upper) and fluorescence (lower) microscopy showed only GFP expression in the area of infiltration (Figure 9**f**, 9**g**, 9**h**).

*MYB110a* was transiently over-expressed in the petals of a white-petalled individual (21-05-09e) of the F_2_ backcross population. *Agrobacterium* strain GV3101, carrying a plasmid (pHEX2) harbouring *35S*::*MYB110a*, was syringed into a small cut in the petal edge. In the area surrounding the site of infiltration, over-expression of *MYB110a* complemented the white phenotype and restored the ability to synthesise anthocyanin in petals (Figures 9a, 9b). Microscopy of a petal demonstrating transient expression (Figure 9c) showed that anthocyanin pigmentation varied in concentration within the pigmented area (Figure 9d) and was confined within individual cells (Figure 9e). Transient over-expression of green fluorescent protein (GFP) in petals showed that *Agrobacterium* alone does not elicit an anthocyanin response in white petals (Figure 9f, 9g, 9h). The luciferase assay and the complementation of white petal phenotype by over-expression of *MYB110a* demonstrate the ability of this transcription factor to activate and regulate the anthocyanin biosynthetic pathway in petals of the *Actinidia* hybrid population.

## Discussion

Previous studies have concentrated on the characterization of red colour in the fruit of *Actinidia* species [33,34]. In those studies three cyanidin-based and two delphinidin-based anthocyanins were identified in fruit of six *Actinidia* species. While the cyanidin compounds were found in all the species examined, only two taxa, *A. melanandra* and *A. arguta* var. *purpurea,* contained the delphinidin-based compounds: delphinidin 3-*O*-(xylosyl)galactoside and delphinidin 3-*O*-galactoside. They were not found in either *A. chinensis* or *A. eriantha*. In our study we found that both delphinidin compounds were present in the petals in a proportion of the progeny genotypes within three of the four families resulting from a backcross between *A. eriantha* and *A. chinensis* var. *chinensis*. Cyanidin and delphinidin are produced through different branches of the anthocyanin pathways, from the action of either the flavonoid 3'-hydroxylase (F3'H) or flavonoid 3'5'-hydroxylase (F3'5'H), so our results suggest that both pathways are activated in the flowers of the *Actinidia* backcross population studied here [34].

Anthocyanin colour perception in plant tissues is affected by the ability of the anthocyanins to undergo changes in chemical form resulting from their interactions with metal ions, other anthocyanin molecules (self-association), or other unrelated compounds (co-pigmentation). These associations may cause a change in hue, or an increase in colour intensity [35,36]. A wide range of potential co-pigment compounds have been reported, but by far the most common are the colourless flavonol and flavone types of flavonoids. Flavonols were found in the petal samples examined here, with three of the four flavonols identified, quercetin-rutinoside, quercetin-glucoside and kaempferol-glucoside, being present in all samples. While kaempferol-rutinoside was present in 60 of the 134 samples, it was only found in small concentrations, and its presence or absence did not correlate with the perceived hue of the petals. Neither was the presence or absence of delphinidin compounds aligned with perceived hue. This can be seen from the two genotypes 21-07-11a and 21-09-13d, where delphinidin was not present in either genotype but petals of 21-09-13d appeared to have a blue tinge (Figure 2). Therefore we conclude that the hue variation observed in the different genotypes cannot be explained by the combinations of anthocyanin or flavonol types recorded. The differences in depth of colour seen in the petals corresponded to the total anthocyanin concentration.

This study indicates that anthocyanin and flavonol biosynthesis in *Actinidia* is a complex and highly regulated process. The flavonol kaempferol and the anthocyanin pelargonidin share the same dihydrokaempferol (DHK) precursor. However, while kaempferol was present in the petals, the DHK was not apparently utilised by dihydroflavonol 4-reductase (DFR) and anthocyanidin synthase (ANS) to produce pelargonidin, as this compound was not detected. Rather, the biosynthetic route to anthocyanins proceeded by the action of F3'H and F3'5'H on the precursors prior to their conversion by DFR and ANS to cyanidin (3'4'-hydroxylation) and delphinidin (3'4'5'-hydroxylation). The presence of F3'5'H activity, however, did not result in the production of the flavonol myricetin, which also has 3'4'5'-hydroxylation. The apparent channelling of substrate into flavonols and anthocyanins with varying patterns of hydroxylation, despite the presence of the F3'H and F3'5'H, has also been observed in other species [2]. It could reflect different developmental timings for the production of flavonols and anthocyanins, metabolic channelling within enzyme complexes, or particular substrate specificity of biosynthetic enzymes such as DFR. The DFR of genera such as *Petunia* and *Cymbidium* have been shown to have only weak activity with DHK, resulting in the near absence of pelargonidin-based anthocyanins in these genera [37].

In general, flavonoids with a free hydroxyl group at the C-3 position of the heterocyclic ring are unstable under physiological conditions, and are therefore typically found as their glycosylated forms. Unlike the case in many plants, differential glycosylation occurs at the C-3 position for flavonols and anthocyanins of *Actinidia*. The flavonols identified were glucosides and rutinosides (resulting from addition of rhamnose to the glucose), while the main anthocyanins were galactosides and xylosyl-galactosides. Only low concentrations of anthocyanin-glucosides were found, and anthocyanin-rutinosides were not detected.

Three R2R3-MYB genes have been identified for *Actinidia* that show the conserved amino acid sequences of activators of anthocyanin biosynthesis – *MYB10, MYB110a* and *MYB110b*. *MYB110a* is thought likely to be the major gene defining petal colour as it was expressed at high levels in red but not white petals, and *MYB110b* expression was not detected at all. Expression for *MYB110a* (and to a lesser extent, *MYB10*) was highest before full bloom, and declined as the flower expanded. This pattern occurs with anthocyanin-related MYBs controlling other floral phenotypes, eg. petunia [11]. The white-petalled phenotype of flowers was complemented through introduction of *MYB110a*, confirming its identity as an anthocyanin regulator. Furthermore, specific gene markers were obtained that linked different alleles of *MYB110a* to the presence of white versus red petals. The results show that *MYB110a* is the agent of specificity of anthocyanin expression in the flower petals while *MYB110b* is probably a tightly linked, but not expressed, relative of *MYB110a*. Fruit flesh and flower ovary and filament colour must be under the control of other R2R3-MYB gene family members, or alleles, as these did not segregate with *MYB110a*. Future work will concentrate on identifying fruit-specific R2R3-MYB markers for use in marker assisted breeding.

In transient assays, adding exogenous bHLH (*AtTT8*) had surprisingly little effect on the activation of the *F3GT1* and *AtDFR* promoters by *MYB110a*. However, it is unlikely that *MYB110a* functions without a bHLH. Instead *MYB110a* may be more efficient at interacting with tobacco bHLHs already present in the transiently expressing cell, and this could be determined using hairpins to tobacco endogenous bHLH genes. Although the bHLH is thought to be the main component that links with the different WDR, R2R3- and R3-MYB proteins in the MBW regulatory complex, variation in bHLH expression is unlikely to provide for tissue-specific anthocyanin production. The results in our study of *Actinidia* support the proposal that variation in R2R3-MYB activator function and expression is the key determinant of spatial and temporal patterning of anthocyanin production in most plant species [15,16].

## Conclusions

We have shown that the red petal phenotype in the interspecific *Actinidia* population is the result of a mixture of anthocyanins, with Cy-gal present in the greatest concentration, and with Cy-xylgal and Cy-glu also present in significant amounts, and in various combinations, in individual genotypes. Other anthocyanins were present in minor quantities in different genotypes. Delphinidin was recorded as both delphinidin 3-*O*-(xylosyl)galactoside (dp-xylgal) and delphinidin 3-*O*-galactoside (dp-gal) and could be considered in parent choice for breeding populations for colour variation in fruit. Red or white petal colour in *A. chinensis* x *A. eriantha* hybrids is determined by *MYB110a*.

## Methods

### Plant material and DNA extraction

Four interspecific backcross families between *A. chinensis* var. *chinensis* and *A. eriantha* were created as detailed in Additional file 1. The population was grown at the Plant & Food Research Te Puke Research Centre, Bay of Plenty, New Zealand. At budbreak, leaf tissue was taken from each genotype, held at 4°C for 24 h, and stored at −80°C until required. DNA was extracted from leaves about 1 cm in length from all progeny, and as many of the parents of the crosses as were available. The sample was ground to powder in liquid nitrogen before being processed through a DNeasy Plant Mini Kit (Qiagen™) according to the manufacturer’s instructions. The final eluate was 200 μl in volume. Five μl of a one in ten dilution of this eluate was used in each PCR reaction.

With the goal of a usable genetic marker in *Actinidia*, the female and male parents of an F_1_*Actinidia* genetic mapping population [30] were also included. The female parent of the mapping population had white flowers and red colour in the fruit, while the male parent carried white flowers and no evidence of red flesh in male line siblings. The male parent of the mapping population was also the male parent of all four families in this study.

### Phenotyping of floral structures and fruit

In October 2009, flowers from 275 backcross hybrid seedlings were scored for the presence of red colour in their petals. Two flower buds from each of 101 female seedlings were dissected and scored for the presence of red colour in the pericarp tissue of the ovary. Flowers from three of the four female F_1_ hybrid parents of the backcross population were also scored for these traits (the fourth parent, 11-06-15c, produced no flowers in 2009). Flowers of the male *A. chinensis* parent involved in the backcross were scored for petal colour. The sex of each seedling was recorded, based on the morphology of the flowers. In 2010, ripe fruit from 88 female vines were screened for the presence of red colour in the fruit flesh.

### Candidate gene identification. Microsatellite identification and primer design

The candidate genes, MYBs, were identified in the Plant & Food Research *Actinidia* expressed sequence tag (EST) database (available in GenBank, FG527909, FG403522).

Exon and intron sequence data were examined to identify dinucleotide microsatellites that would be suitable for marker identification of the MYB genes. Primer pair sequences were chosen which gave a theoretical PCR product size between 200 and 250 bp, with an annealing temperature between 58°C and 60°C, and with a GC content of approximately 50%. As the *MYB110a* gene closely resembled the neighbouring *MYB110b* gene in 5’ sequence, the primers were designed to amplify a sequence at the 3’ end of the *MYB110a* gene. Primer 3 software was used to identify and reduce the incidence of hairpins and primer dimers. One of the primers of the pair was located in an intron sequence while the other was designed within the exon to facilitate marker specificity.

### Polymerase chain reaction, electrophoresis, and analysis

The primer pairs were screened for PCR amplification in available parents and progeny in samples of the four families of the interspecific cross. A reaction mix of 15 μl containing 1 × PCR buffer (20 mM Tris–HCl, 50 mM KCl), MgCl_2_ 5 mM (the buffer and MgCl_2_ were those supplied with the polymerase), 0.2 mM each of dNTPs, 4.5 pmol of each primer, and 1.25 units of Platinum Taq polymerase (Invitrogen), was prepared for each DNA sample. About 12.5 ng of genomic DNA was added in 5 μl to bring the total PCR volume to 20 μl. PCRs were performed in a Techne™ TC-412 thermal cycler with a single cycle of 94°C for 3 min preceding 35 cycles of denaturing at 94°C for 30 sec, annealing for 30 sec, and elongation at 72°C for 1 min. PCR reactions were carried out with primers that were labelled with 6FAM, and prepared for analysis. The allelic content of each genotype was determined by capillary electrophoresis in an ABI Prism® 3100 Genetic Analyzer (Filter Set D, ROX™ size standard), and analyzed with GeneMapper™ Software Version 3.7 (Applied Biosystems).

### Real time qPCR expression analysis

Petals were collected from individual genotypes, representing both red and white petal types, at late calyx split and full bloom. The samples were immediately frozen in liquid nitrogen in the field, and held at −80°C.

RNA was isolated from petals [31] and DNAse-treated (DNA-free:Ambion. http://www.ambion.com/). Reverse transcription was performed using oligo(dT) according to the manufacturer’s protocol (Transcriptor: Roche Diagnostics, http://www.roche.com/).

Genes were identified by BLAST with genes of known function in an *Actinidia* EST database [26]. Phylogeny of *MYB* proteins were generated from alignments (using Clustal W) and molecular evolutionary analysis conducted using MEGA version 4.0.2 [38] (using minimum evolution phylogeny test and 1000 bootstrap replicates).

Gene-specific primers were designed using Vector NTI 9.0.0 (http://www.invitrogen.com) and are summarised in a supplementary table (Additional file 5). Quantitative real-time PCR was carried out using the LightCycler 480 System (Roche Diagnostics), reactions were performed in quadruplicate and a non-template control was included in each run. Thermal cycling conditions were 95°C for five min, then 40 cycles of 95°C for 10 sec, 60°C for 10 sec, and 72°C for 15 sec, followed by a melting temperature cycle, with constant fluorescence data acquisition from 65°C to 95°C. The data were analysed using the Target/Reference ratio calculated with the LightCycler® 480 software 1.5, enabling a comparison of the level of expression of two different genes normalised to two reference genes: actin and subunit protein phosphatase 2A (PP2A) [31]. To confirm primer specificity, the qPCR product was sequenced (The Allan Wilson Centre: http://www.allanwilsoncentre.ac.nz).

### Complementation study by transient expression in petals and dual luciferase assay

DNA constructs have been created to over-express the MYB/bHLH in tobacco (*Nicotiana benthamiana*) leaves and *Actinidia* petals: pHex2-*MYB110a*, pHex2-*AtMYB75*, pHex2-*AtTT8* and 1Kb promoter sequence of *F3GT1* inserted into pGreen 0800-LUC for a dual luciferase assay [39], or the *AtDFR* promoter fused to LUC as described in Espley et al. [32]. *Agrobacterium tumefaciens* GV310(MP90) were transformed with the different constructs. *Agrobacterium* cultures were resuspended in infiltration buffer prior to infiltration [39]. Flowers from 21-05-09e, a white-flowered genotype, were selected for transient expression studies. A small incision in the petal was required to allow infiltration of the *Agrobacterium* suspension and after 3 days it was possible to assess the red pigmentation induced by the over-expressed MYB.

Promoter activation, dual luciferase assay, was performed on *N. benthamiana* leaves (four replicates per treatment). The promoter was co-infiltrated with the different transcription factors tested. The *F3GT1* promoter sequence was fused to the luciferase reporter gene *LUC*, while in the same construct another luciferase r-gene *REN*, under the control of a 35S promoter, was used as an internal control and as indicator of the extent of the transient expression. Three days after inoculation, firefly luciferase and *Renilla* luciferase were assayed using the dual luciferase assay reagent (Promega, Madison, WI, USA). Activity is expressed as a ratio of *LUC* to *REN* activity [39].

### Anthocyanin identity and quantity analyses

A single bud at the early petal emergence stage of development was collected from each flowering genotype. Each bud was dissected within 1 h of collection. An inner petal was selected to reduce the possibility that colour may have faded during exposure to light. As anthocyanin production is sensitive to environmental factors, all measurements were made in a single flowering season from plants grown in one area. Anthocyanins were extracted from petals by soaking in a solvent mixture composed of ethanol/water/formic acid (80/20/1). Screw capped vials were prepared containing 1.0 mL solvent and 50 μg of quercetin (internal standard). Weighed portions of petals were added to the vials, which were then capped, frozen at −20°C and transported to Plant & Food Research, Palmerston North, for analysis.

Ultra High Performance Liquid Chromatography (UHPLC) was used to separate and measure the anthocyanins present in extracts of petal tissue. The UHPLC system used was a Dionex Ultimate® 3000 Rapid Separation LC system equipped with a binary pump (HPR-3400RS), autosampler (WPS-3000RS), column compartment (TCC-3000RS), and a diode array detector (DAD-3000RS). The analytical column employed was a Zorbax SB-C18 HD 2.1 x 150 mm, 1.8 μm (Agilent Technologies, Santa Clara, United States) maintained at 45°C. A binary solvent programme was used with Solvent A (formic acid/MQ water 5:95) and Solvent B (acetonitrile) at a flow of 450 μL/min. The initial solvent composition was 5%A 95%B until 0.5 minutes, then changed to 70%A 30%B at 3.5 min, 60%A 40%B at 9.0 min, and 20%A 80%B at 9.5 min. After a 2-min hold at 20%A 80%B, the composition was returned to 95%A 5%B ready for the next injection. Total UHPLC analysis time was 13 min per sample. All solvent gradients were linear. The injection volume was 0.5 μL. Spectral data (260–600 nm) were collected for the entire analysis, and the anthocyanin components were identified and quantified from chromatograms extracted at 530 nm. External calibration curves were constructed for cyanidin 3-*O*-glucoside obtained from Extrasynthese, (Genay, France). All anthocyanin concentrations were calculated as cyanidin 3-*O*-glucoside equivalents on a fresh weight basis. Individual anthocyanins were identified based on similarity of retention times and spectral properties to authentic compounds (cyanidin 3-*O*-glucoside, cyanidin 3-*O*-galactoside) when available, or to anthocyanins isolated during previous research [33]. Chromatographic data were collected and manipulated using the Chromeleon® Chromatography Management System version 6.8 (Dionex Corporation).

### Statistical analysis

We used ‘heatmap.2’ function of the ‘gplots’ package in R [40] to construct heatmaps of pigment data for two of the backcross populations with larger family sizes. A heatmap is a graphical display of data which uses colours to represent the numerical values. For example, smaller values can be assigned cooler blue tones while the larger values can tend towards hotter orange and red colours. Heatmaps are often used to encode and display multivariate data where several variables are measured on a set of entities (e.g. vines) so that the data structure is a matrix of columns and rows. The colours are automatically scaled for each variable of the data matrix, but this can be disabled if the data have already been normalised. For multivariate data heatmaps can also rearrange rows and columns so that similar entities and variables are grouped together and each is represented by a dendrogram.

## Competing interests

We do not have any competing interests to report.

## Authors’ contributions

LGF conceived the genotyping study, carried out SSR discovery, marker polymorphism discovery and population genotyping and analyses, drafted the manuscript, and coordinated the final version. AGS carried out phenotyping, and discovered the 1:1 segregation ratio for flower colour in the backcross population. AGS and HEM bred the hybrid population. JKD assisted with phenotyping. GKT and EH participated in population genotyping; GKT also assisted in preparing the manuscript. PMD performed genotyping analyses and map construction. HNDS assisted in the design of the study and performed the statistical analysis, and wrote the statistical methods section. TKM carried out the quantification and identification of anthocyanins and flavonols and wrote the method for the manuscript. MM sequenced relevant portions of the genome, carried out qPCR, and assisted with manuscript preparation. ACA advised study directions, carried out transient assays and had input in manuscript preparation. KMD contributed to revision of the manuscript draft. MAM assisted with sample collection and manuscript preparation. All authors read and approved the final manuscript.

## Supplementary Material

Additional file 1 **Pedigree of the four *Actinidia *families that show segregation of petal colour.** All the parents involved in creating the four F_2_ backcross families that demonstrated segregation of petal colour were from two *Actinidia* taxa, *A. chinensis* var. *chinensis* and *A*. *eriantha*. The parents of each generation are shown. The female parent is indicated by a pink blaze, and the male parent by blue. *A. eriantha* contributed the red petal phenotype to the segregating population. Click here for file

Additional file 2 **Alleles of *MYB110a *carried by the parents of the four F**_**2 **_**backcross *Actinidia *families.** The alleles of *MYB110a* that are present in all parents of the backcross are represented by the sizes of the PCR products amplified by the primers of the marker Ke923. Where experimental data could not be generated, through loss of the parental genotype, the sizes of the alleles were inferred from the parents and progeny of that genotype. Click here for file

Additional file 3 **Red expression in petals, anther filaments and ovaries in the flowers of four F2 backcross hybrid *Actinidia *families.** Ovary colour was independent of petal colour. Stamen filament colour was also independent of petal and ovary colour. Red filaments were found with both red or white petals, and red or green ovaries, in all combinations in female progeny, and with red or white petals in males. Click here for file

Additional file 4 **Anthocyanin and flavonoid analysis data.** Identities and concentrations of anthocyanins and flavonols extracted from red petals of flowers of four inter-related F_2_ backcross *Actinidia* families. Click here for file

Additional file 5 **Gene-specific primer sequences.** Quantitative real-time PCR was carried out with gene-specific primers that were designed using Vector NTI 9.0.0. PCR primers of marker Ke923 specific for *MYB110a*, and marker Ke701 specific for *MYB110b* were designed by LGF. (DOC 32 kb) Click here for file
