# Supplementary figures and images for: An R2R3 MYB transcription factor determines red petal colour in an Actinidia (kiwifruit) hybrid population
Source: BMC Genomics. 2013 Jan 16;14:28. doi: 10.1186/1471-2164-14-28 (PMC3618344; doi:10.1186/1471-2164-14-28)

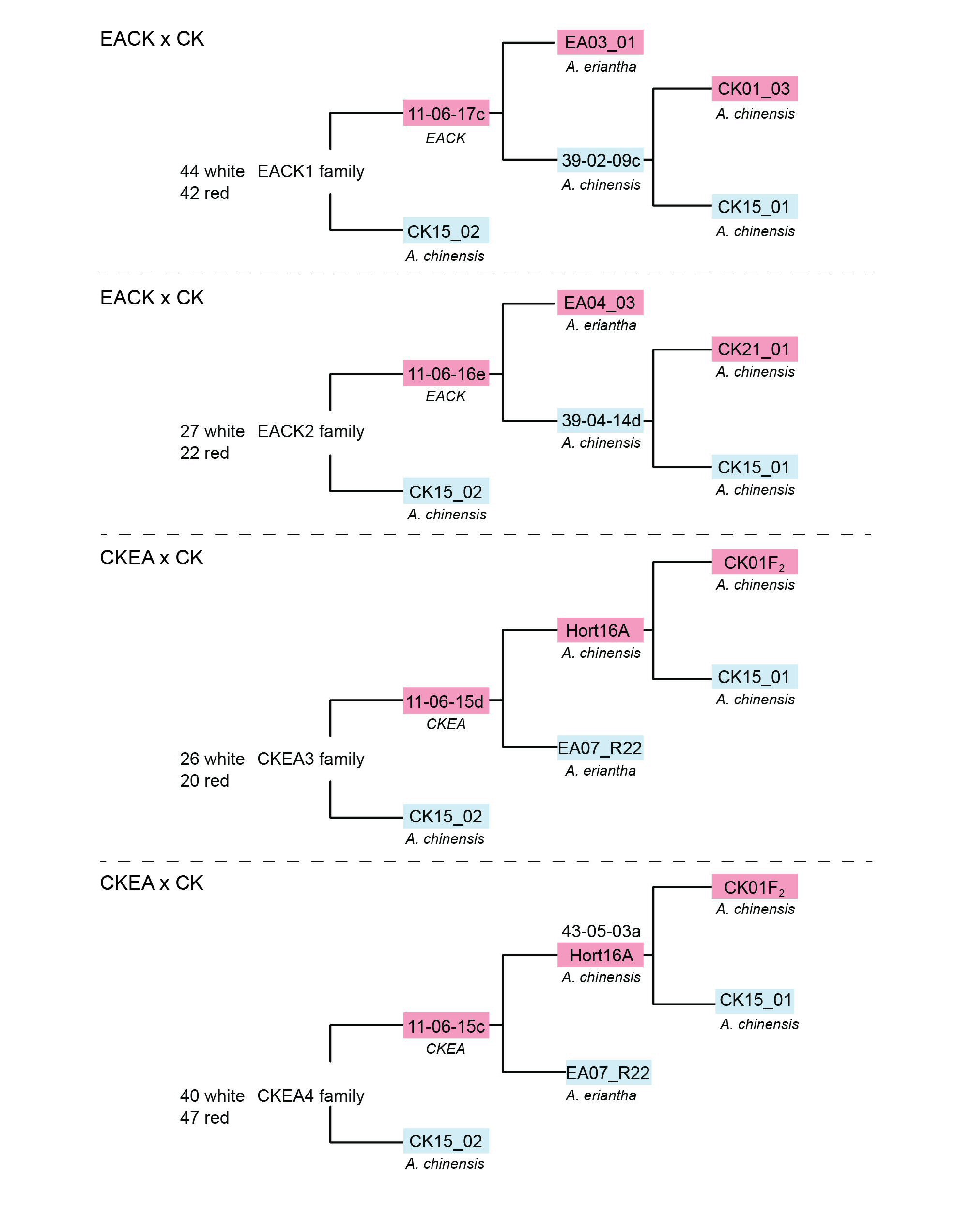

Supplement: Additional file 1 — Pedigree of the four Actinidia families that show segregation of petal colour. All the parents involved in creating the four F2 backcross families that demonstrated segregation of petal colour were from two Actinidia taxa, A. chinensis var. chinensis and A. eriantha. The parents of each generation are shown. The female parent is indicated by a pink blaze, and the male parent by blue. A. eriantha contributed the red petal phenotype to the segregating population. [file 1471-2164-14-28-S1.jpeg]

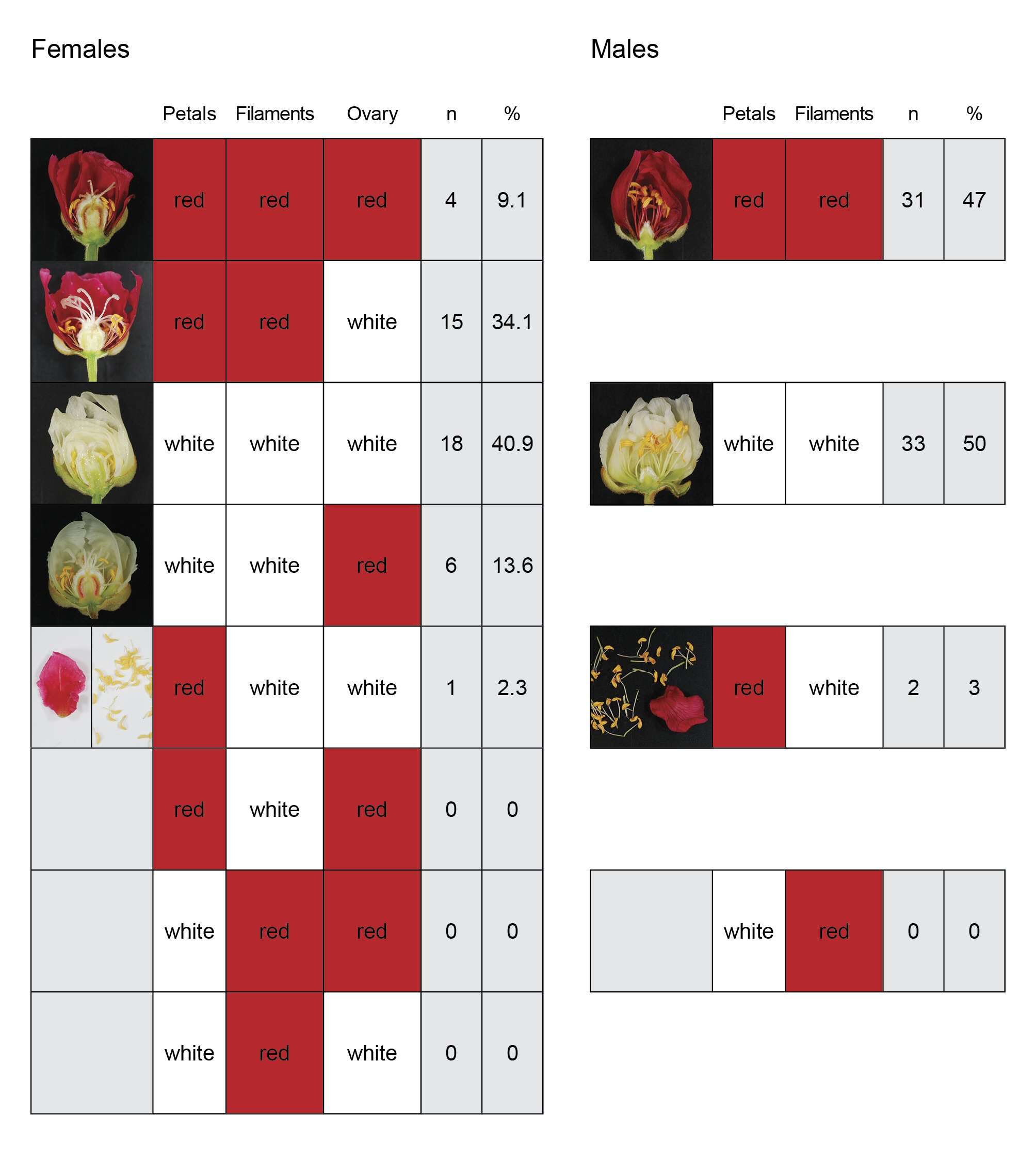

Supplement: Additional file 3 — Red expression in petals, anther filaments and ovaries in the flowers of four F2 backcross hybrid Actinidia families. Ovary colour was independent of petal colour. Stamen filament colour was also independent of petal and ovary colour. Red filaments were found with both red or white petals, and red or green ovaries, in all combinations in female progeny, and with red or white petals in males. [file 1471-2164-14-28-S3.jpeg]
