# Supplementary material for: An R2R3 MYB transcription factor determines red petal colour in an Actinidia (kiwifruit) hybrid population
Source: BMC Genomics. 2013 Jan 16;14:28. doi: 10.1186/1471-2164-14-28 (PMC3618344; doi:10.1186/1471-2164-14-28)
Supplement: Additional file 2 — Alleles of MYB110a carried by the parents of the four F2 backcross Actinidia families. The alleles of MYB110a that are present in all parents of the backcross are represented by the sizes of the PCR products amplified by the primers of the marker Ke923. Where experimental data could not be generated, through loss of the parental genotype, the sizes of the alleles were inferred from the parents and progeny of that genotype. [file 1471-2164-14-28-S2.docx]

209 359

209 361

EA03_01

228 426

228 410

39-02-09c

nd 413

nd 410

CK01_03

228 426

228 426

CK15_01

228 410

209 361

11-06-17c

Family 1 (EACK x CK)

228 427

228 426

CK15_02

228 426

209 361

228 427

228 410

228 426

228 410

228 427

209 361

228 426

209 359

228 427

228 426

228 426

228 426

228 427

209 359

228 427

228 426

CK15_02

Family 3 (CKEA x CK)

228 426

209 359

11-06-15d

228 408

228 406

CK01F_2_

228 426

228 426

CK15_01

209 nd

209 nd

EA07_R22

228 426

228 406

Hort16A

228 426

209 359

228 427

228 432

228 426

228 432

228 427

209 359

228 427

228 426

CK15_02

Family 2 (EACK x CK)

228 432

209 359

11-06-16e

228 432

228 409

CK21_01

228 426

228 426

CK15_01

228 426

228 432

39-04-14d

228 359

209 359

EA04_03

Family 4 (CKEA x CK) had same alleles and pedigree except it had 11-06-15c (full sib of 11-06-15d)
