# Supplementary material for: An R2R3 MYB transcription factor determines red petal colour in an Actinidia (kiwifruit) hybrid population
Source: BMC Genomics. 2013 Jan 16;14:28. doi: 10.1186/1471-2164-14-28 (PMC3618344; doi:10.1186/1471-2164-14-28)
Supplement: Additional file 5 — Gene-specific primer sequences. Quantitative real-time PCR was carried out with gene-specific primers that were designed using Vector NTI 9.0.0. PCR primers of marker Ke923 specific for MYB110a, and marker Ke701 specific for MYB110b were designed by LGF. (DOC 32 kb) [file 1471-2164-14-28-S5.doc]

| Accession no | Gene | Primers | | Efficiency |
| --- | --- | --- | --- | --- |
|  |  | Forward | Reverse |  |
| GU079683 | *F3GT1* | TAGCCAAGCAGAGATCCGCTTCC | CAAGAATCCTTCTGGTAAGTACTGTTTCGA | 1.91 |
| FG527909 | *MYB10* | CTTCCGGGAAGAACATCAAACGA | GGCGGTGACTTGGGGTGG | 1.85 |
| FG403522 | *MYB110* | ATATCTACAAGAGAAGAGCCGATACCCAA | TGGTTGAATGCTATCTGCAATGACAGT | 1.86 |
| FG428757 | *PP2A* | GCAGCACATAATTCCACAGG | TTTCTGAGCCCATAACAGGAG | 1.94 |
| FG440519 | *Actin* | CCAAGGCCAACAGAGAGAAG | GACGGAGGATAGCATGAGGA | 1.84 |
|  |  |  |  |  |
|  |  |  |  | Marker No. |
|  | *MYB110a* | TGACCTATGGAATCTCTTAGGTG | TCCGGTGAATTGAATAGGCA | Ke923 |
|  | *MYB110b* | TGGCACCAAGTACCTTGCAGA | CACATTAAGATAACAGAAGTGCG | Ke701 |
